# Supplementary material for: Intermittent scavenging of storage lesion from stored red blood cells by electrospun nanofibrous sheets enhances their quality and shelf-life
Source: Nat Commun. 2022 Dec 1;13:7394. doi: 10.1038/s41467-022-35269-3 (PMC9712616; doi:10.1038/s41467-022-35269-3)
Supplement: Supplementary file 1 — Supplementary Information [file 41467_2022_35269_MOESM1_ESM.pdf]

Supplementary Information for -

Intermittent scavenging of storage lesion from stored red blood cells by electrospun nanofibrous sheets enhances their quality and shelf-life

Subhashini Pandey[1,2#], Manohar Mahato[1#], Preethem SrInath[1], Utkarsh Bhutani[1], Tanu Jain Goap[1,2], Priusha Ravipati [1], and Praveen Kumar Vemula[1*]

[1] Institute for Stem Cell Science and Regenerative Medicine (inStem), GKVK Post, Bellary Road, Bangalore 560065, Karnataka, India

[2] The University of Trans-Disciplinary Health Sciences and Technology, Attur (post), Yelahanka, Bangalore, 560064 Karnataka, India.

Corresponding author:

*e-mail: praveenv@instem.res.in

#Equal Contribution

This Supplementary Information file include:

Supplementary Figure 1. Scheme for the synthesis of charged polymers, poly-acridine and poly-taurine.
Supplementary Figure 2. A complete blood count of stored RBCs before and after *Tau-Acr*NFS treatment.
Supplementary Figure 3. Scavenging of DAMPs using charged nanofibrous sheets.
Supplementary Figure 4. Single and twice intermittent scavenging of DAMPs using *Tau-Acr*NFS from stored RBCs can reduce the accumulation of DAMPs at 14 days of stored mice RBCs.
Supplementary Figure 5-16. Flow cytometry profiles.
Supplementary Figure 17. Representation of cytokine array blots
Supplementary Table 1. The ion Q1/Q3 transitions and other parameters to quantify PUFAs.
Supplementary Table 2. The optimized operating conditions used for ICP-MS.

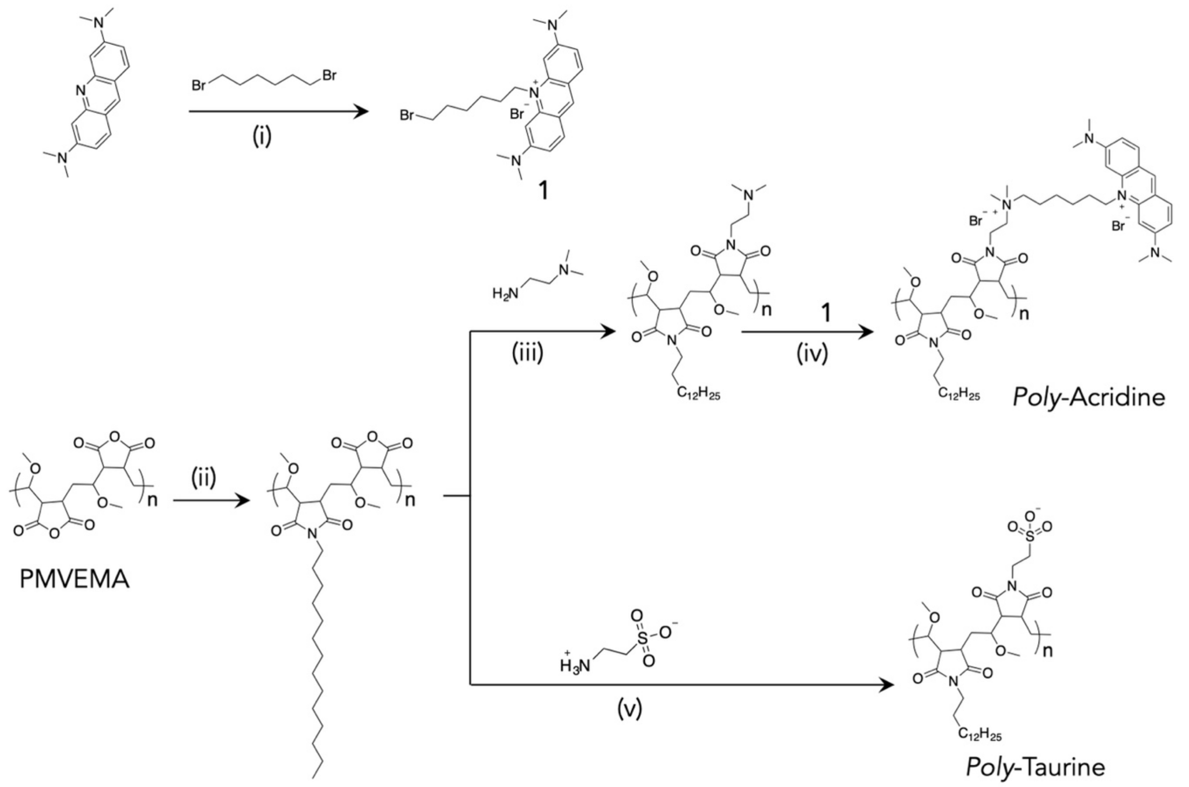

Supplementary Figure 1. Scheme for the synthesis of charged polymers, poly-acridine and poly-taurine. Poly-acridine and poly-taurine polymers were synthesized using solvent phase synthesis. A detailed procedure has been described in Methods. The reaction conditions were as following; (i) anhydrous toluene, 120 ºC, 12 hours; (ii) tetradecyl amine, anhydrous tetrahydrofuran, 80 ºC, 3 hours; (iii) anhydrous tetrahydrofuran, 80 ºC, 12 hours; (iv) anhydrous dimethylformamide, 50 ºC, 16 hours; and (v) anhydrous tetrahydrofuran, 50 ºC, 16 hours.

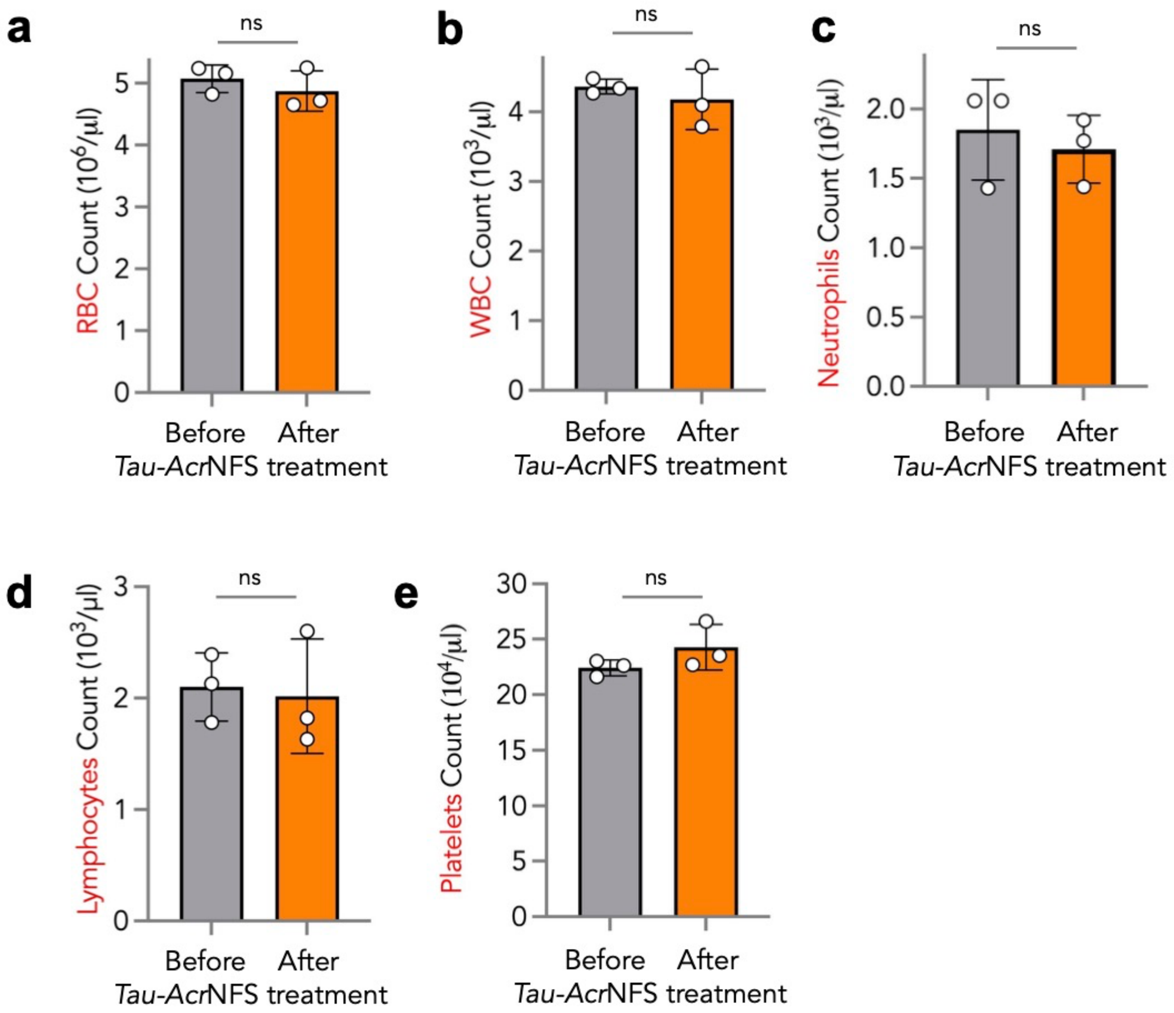

Supplementary Figure 2. A complete blood count of stored RBCs before and after *Tau-Acr*NFS treatment. a-e, Treatment of stored blood with *Tau-Acr*NFS did not cause loss of cells. Quantification of RBCs (**a**), WBCs (**b**), neutrophils (**c**), lymphocytes (**d**), and platelets (**e**) before and after treatment with *Tau-Acr*NFS, Data are mean ± s.d. (n = 3, from independent experiments). For a-e, P values were determined by two-tailed Student's t-test with Welch's correction using GraphPad PRISM 9, ns = not significant. Source data are provided as a Source Data file.

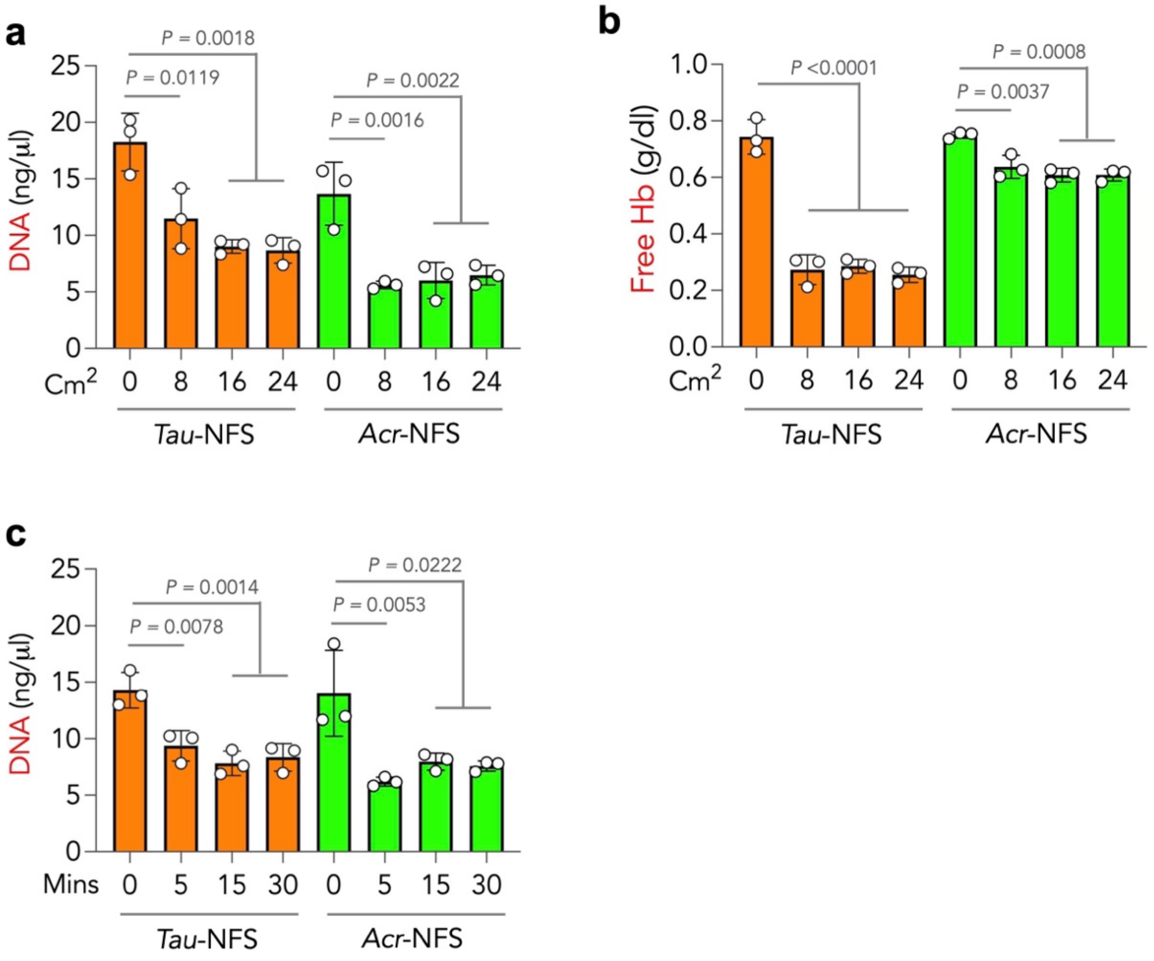

Supplementary Figure 3. Scavenging of DAMPs using charged nanofibrous sheets. a, b. 42 days-stored RBCs were incubated with either *Tau*-NFS or *Acr*-NFS with varying surface area (8, 16, and 24 cm^2). **c**, 42 days-stored RBCs were incubated with either *Tau*-NFS or *Acr*-NFS with varying incubation time (5, 15, and 30 mins). Data are mean ± s.d. (n = 3, from independent experiments); P values were determined by ordinary one-way ANOVA with Tukey's post hoc analysis using GraphPad PRISM 9, and exact P values are indicated. Source data are provided as a Source Data file.

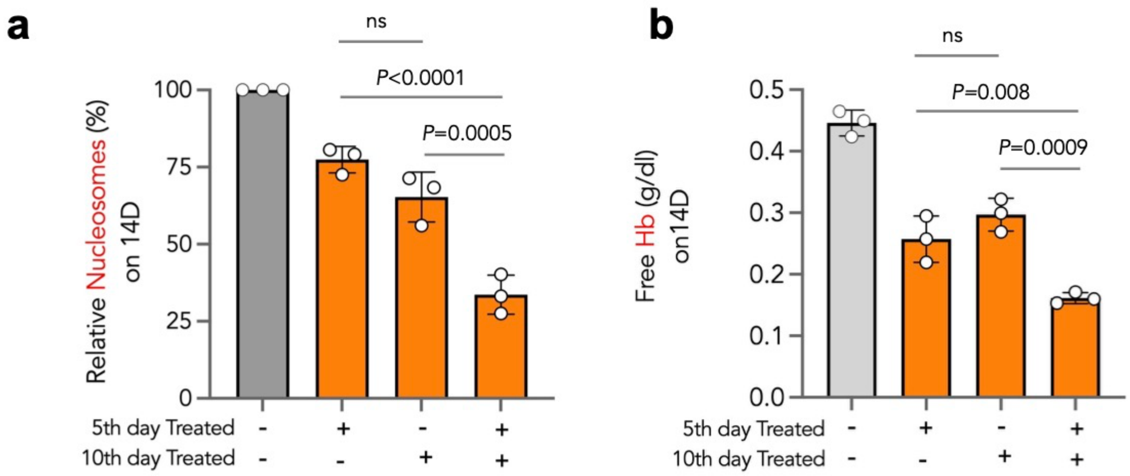

Supplementary Figure 4. Single and twice intermittent scavenging of DAMPs using *Tau-Acr*NFS from stored RBCs can reduce the accumulation of DAMPs at 14 days of stored mice RBCs. Intermittent treatment of stored RBCs twice with *Tau-Acr*NFS on 5th and 10th days significantly reduced the accumulation of DNA (**a**), and Hb (**b**) compared to the samples that were treated only once either on 5th day or 10th day. Data are mean ± s.d. (*n* = 3, from independent experiments); *P* values were determined by ordinary one-way ANOVA with Tukey's post hoc analysis using GraphPad PRISM 9, and exact *P* values are indicated, ns = not significant. Source data are provided as a Source Data file.

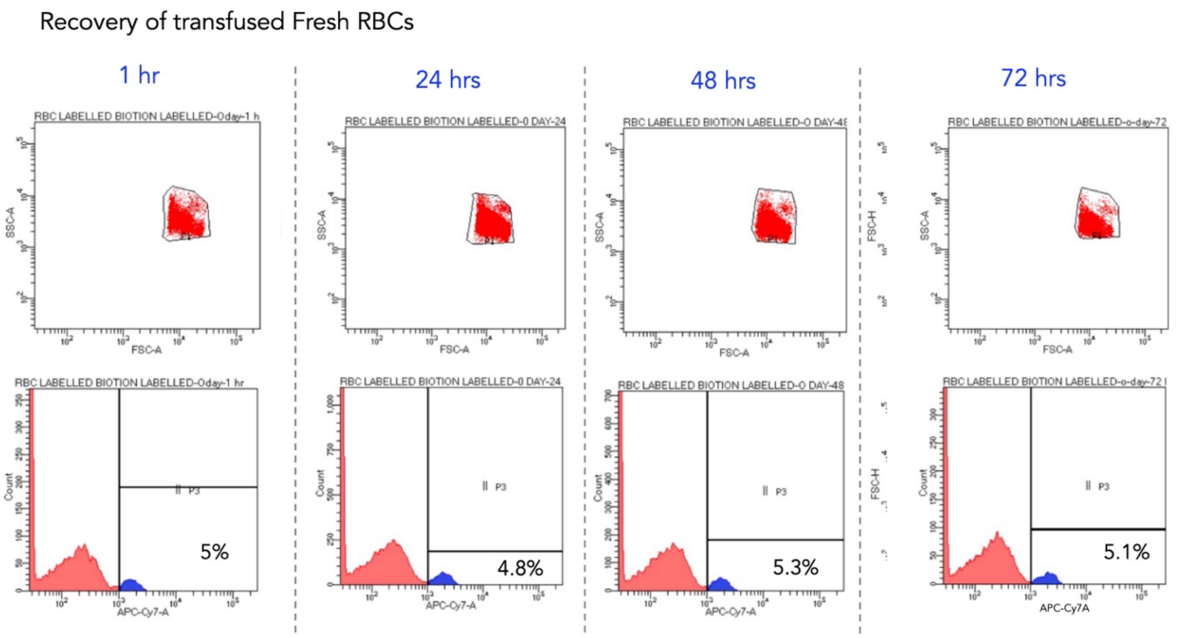

Supplementary Figure 5. Freshly stored biotinylated RBCs were transfused into mice, collected blood at post-transfusion 1, 24, 48 and 72 hrs, and labelled with Streptavidin APC-eFluor™, and quantified via flow cytometry. Flow cytometry profiles indicate that more than 75% of labelled RBCs were in the circulation, which suggests the good quality of RBCs.

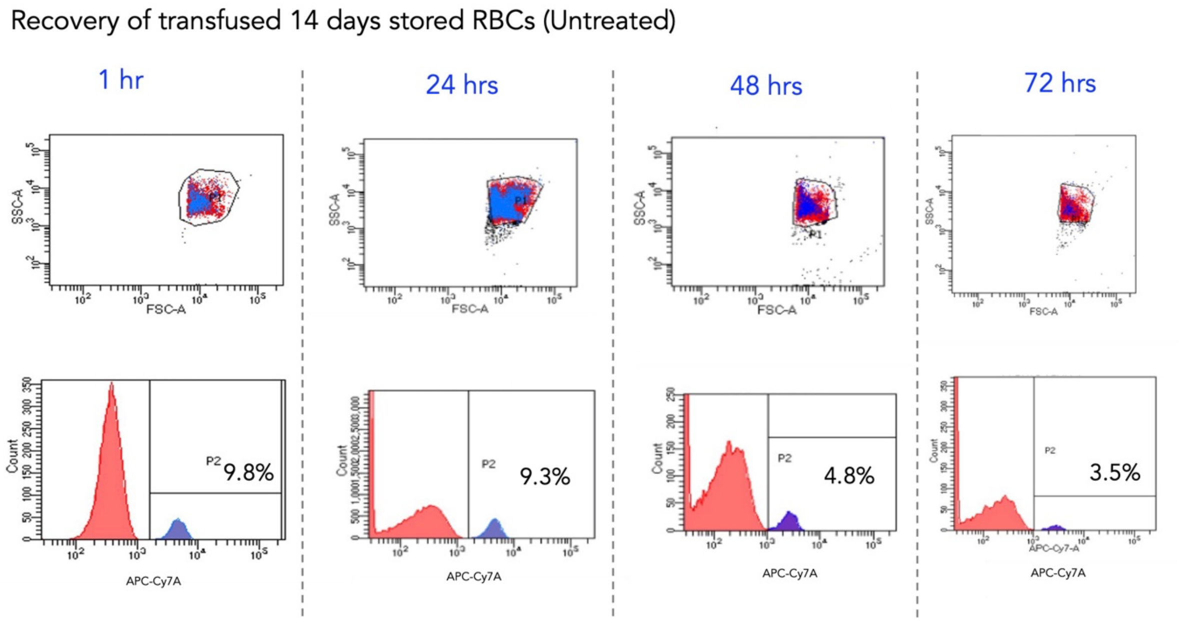

Supplementary Figure 6. Flow cytometry profiles for recovery of transfused untreated 14 days stored RBCs shows that 90% of untreated transfused RBCs were in circulation for 24 hours, which was reduced to <40% at 48 hours suggesting that the quality of stored old RBCs deteriorated significantly.

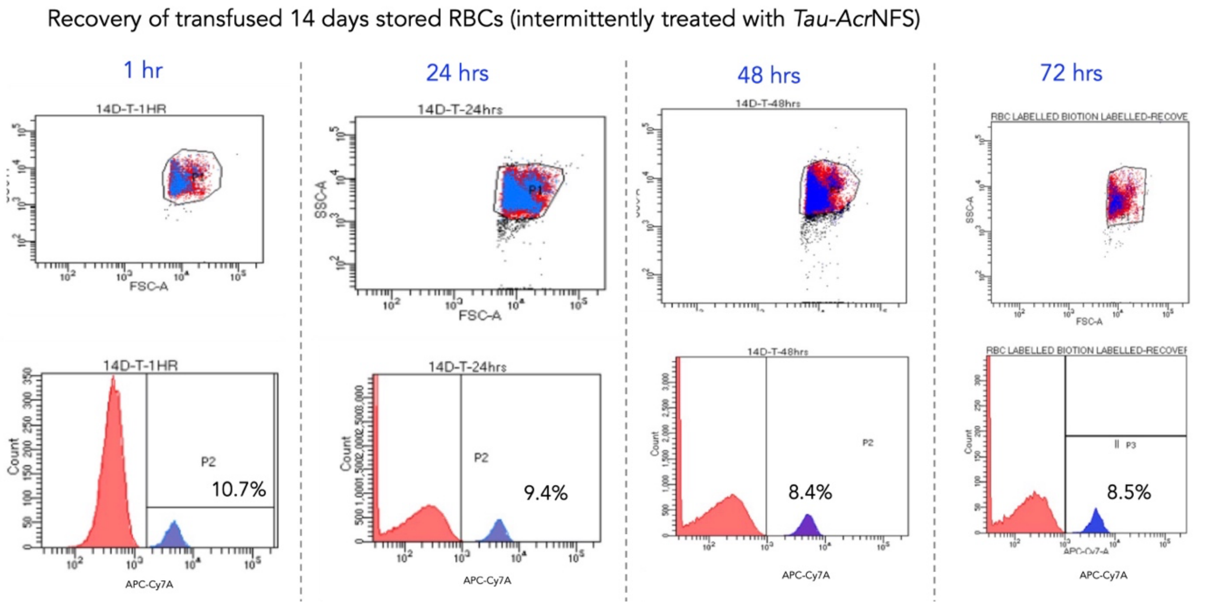

Supplementary Figure 7. Flow cytometry profiles for recovery of transfused 14 days stored RBCs that were treated intermittently with *Tau-Acr*NFS remained >90% in circulation even at 72 hours suggesting that intermittent treatment slows down stored RBC ageing and prevents the clearance.

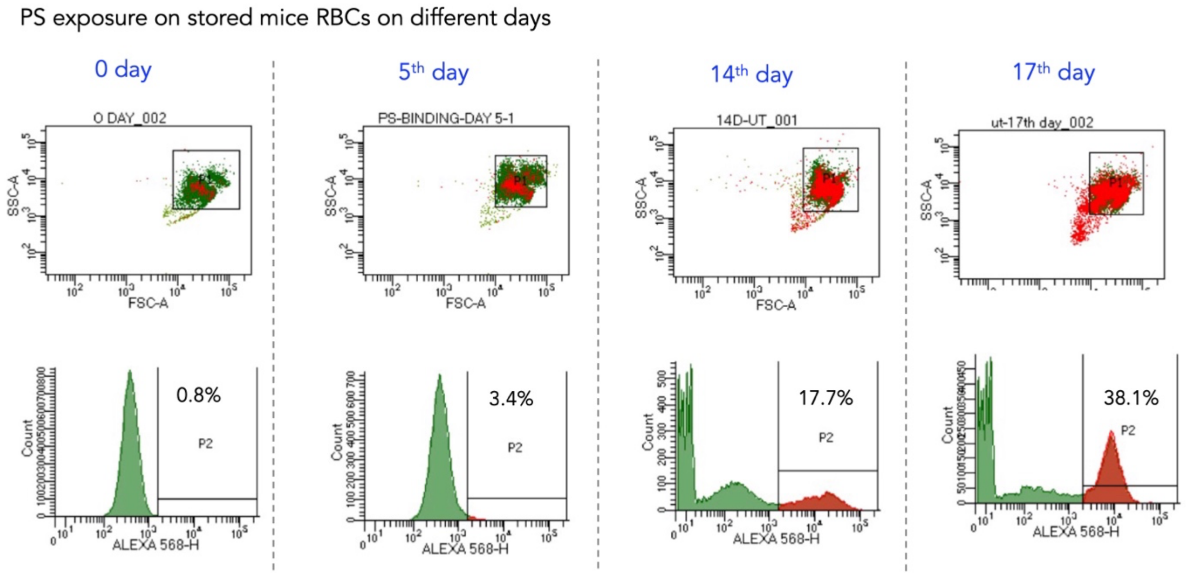

Supplementary Figure 8. Flow cytometry profiles for PS exposed on stored mice RBCs as a function of time. There is an increase of phosphatidylserine (PS) exposure on the outer leaflet of the RBCs membrane during storage, which serves as an ‘eat-me’ signal, thus, RBCs will be eliminated from circulation by phagocytes.

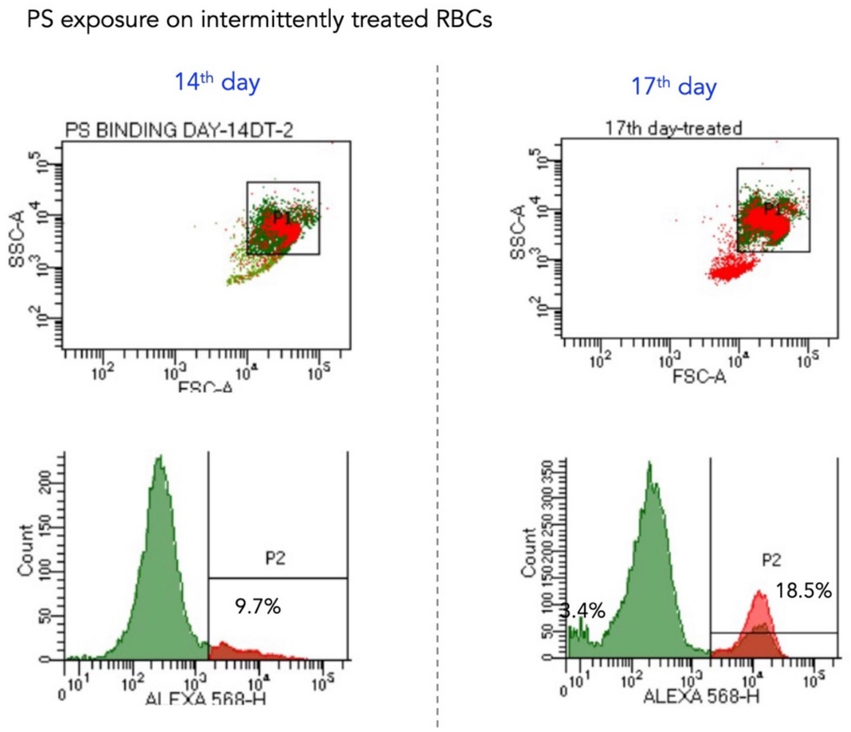

Supplementary Figure 9. Flow cytometry profiles for PS exposed RBCs that were treated with *Tac-Acr*NFS treated intermittently and stored for 14 and 17 days. The treatment group revealed a marked decrease in the PS exposure (%) on the 14th day (~10%) and 17th day (~18%) compared to the untreated group. The low exposure (%) of PS in observed in the treated group.

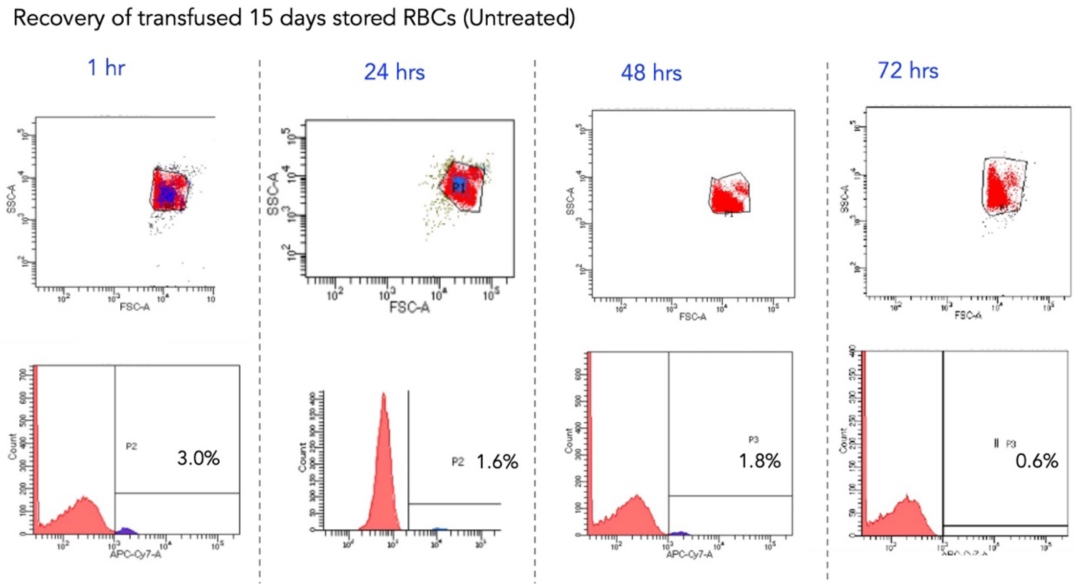

Supplementary Figure 10. Flow cytometry profiles for recovery of transfused untreated 15 days stored RBCs cleared up by the system in 24 hours. The recovery of transfused RBCs were <50% after 24 hours posttransfusion.

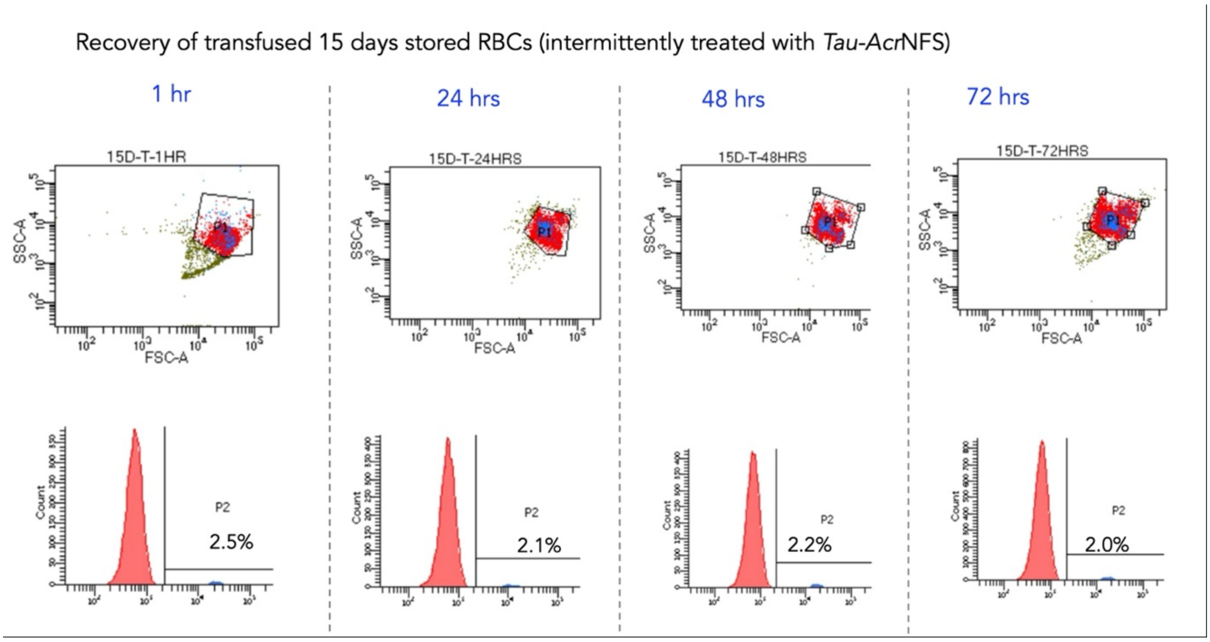

Supplementary Figure 11. Flow cytometry profiles for recovery of transfused 15 days stored RBCs that were treated intermittently with *Tau-Acr*NFS stays in circulation up to 72hrs, suggests that treatment with *Tau-Acr*NFS has enhanced the shelf-life of RBCs.

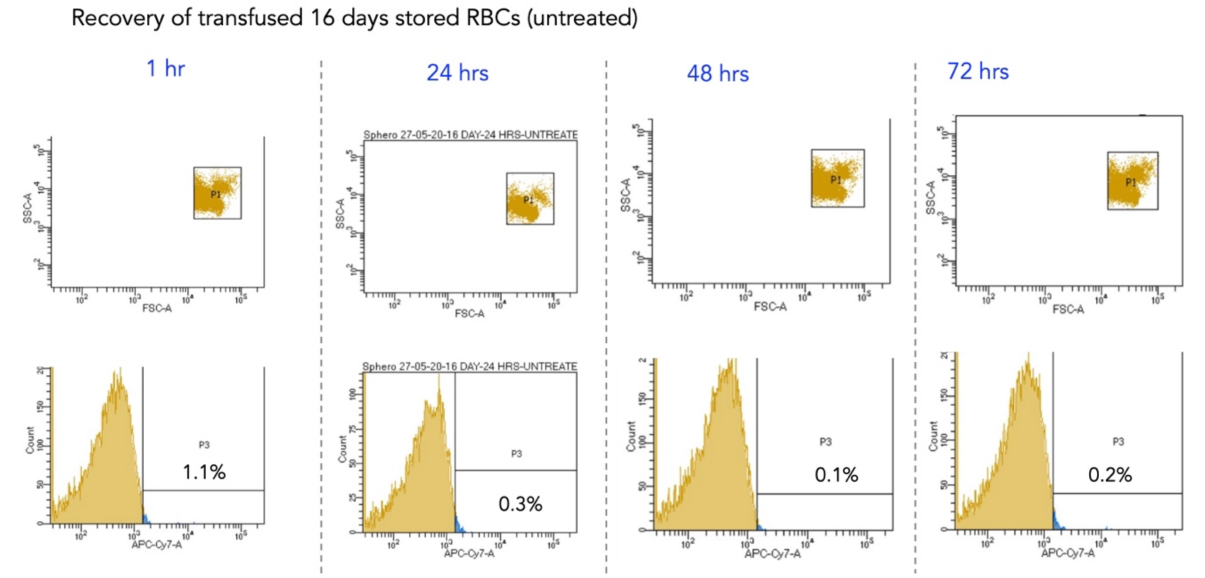

Supplementary Figure 12. Flow cytometry profiles for recovery of transfused untreated 16 days stored RBCs. The recovery of transfused RBCs were <25% after 24 hours posttransfusion.

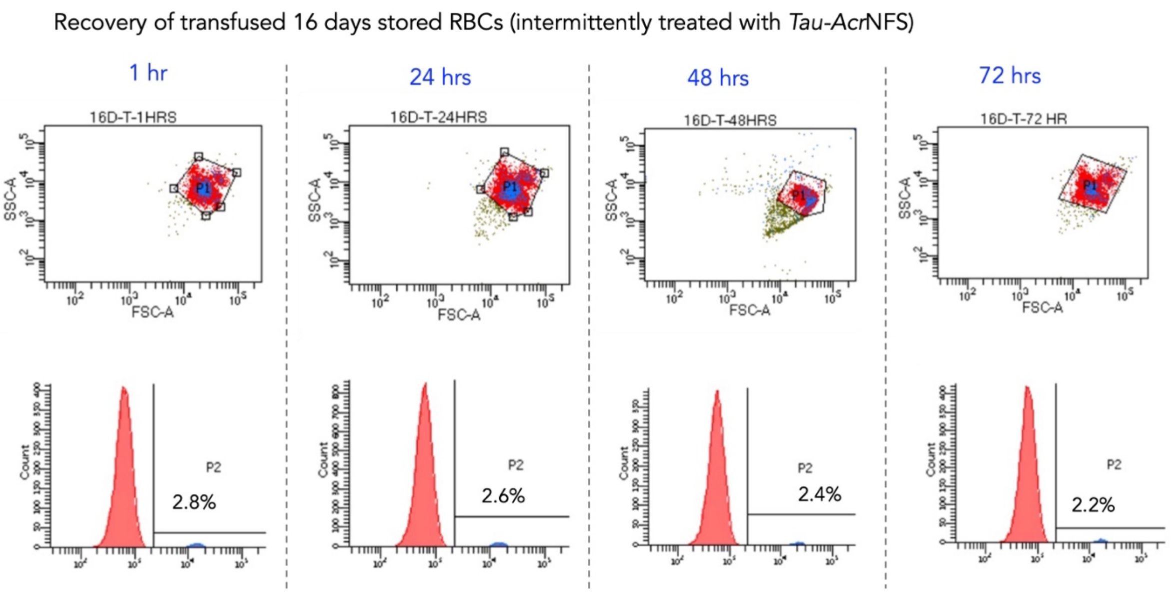

Supplementary Figure 13. Flow cytometry profiles for recovery of transfused 16 days stored RBCs that were treated intermittently with *Tau-Acr*NFS. A >90% of transfused RBCs were in circulation at 72 hours suggests that treatment with *Tau-Acr*NFS has enhanced the shelf-life of RBCs.

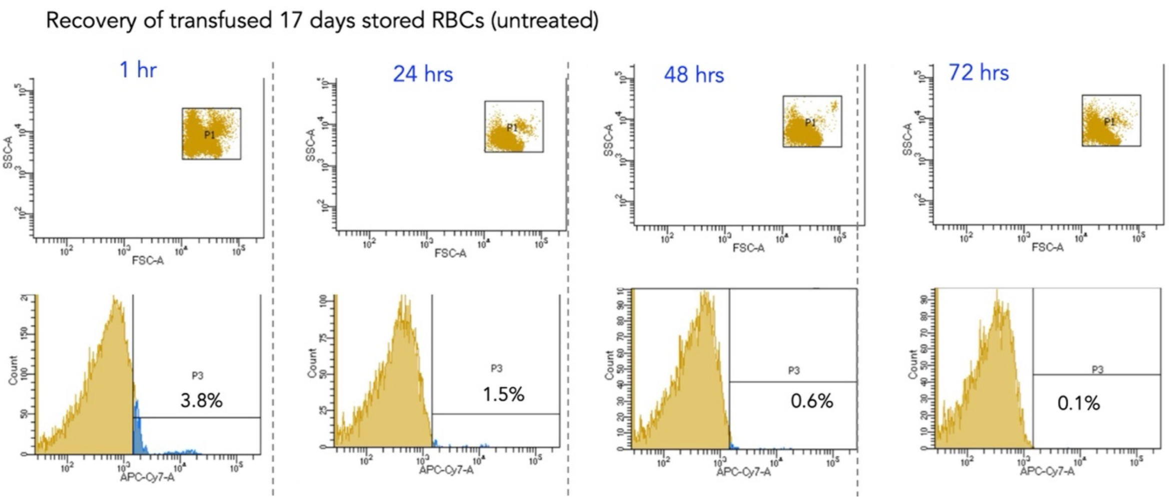

Supplementary Figure 14. Flow cytometry profiles for recovery of transfused untreated 17 days stored RBCs. The recovery of transfused RBCs were <25% after 24 hours posttransfusion.

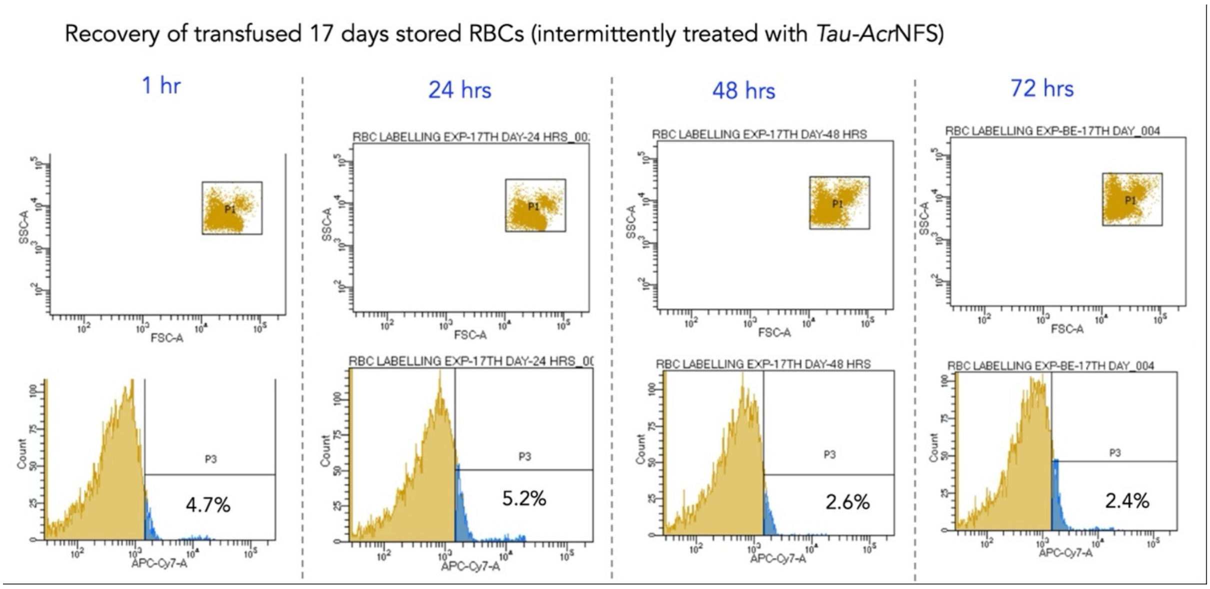

Supplementary Figure 15. Flow cytometry profiles for recovery of transfused 17 days stored RBCs that were treated intermittently with *Tau-Acr*NFS. A >80% of transfused RBCs were in circulation at 24 hours suggests that treatment with *Tau-Acr*NFS has enhanced the shelf-life of RBCs.

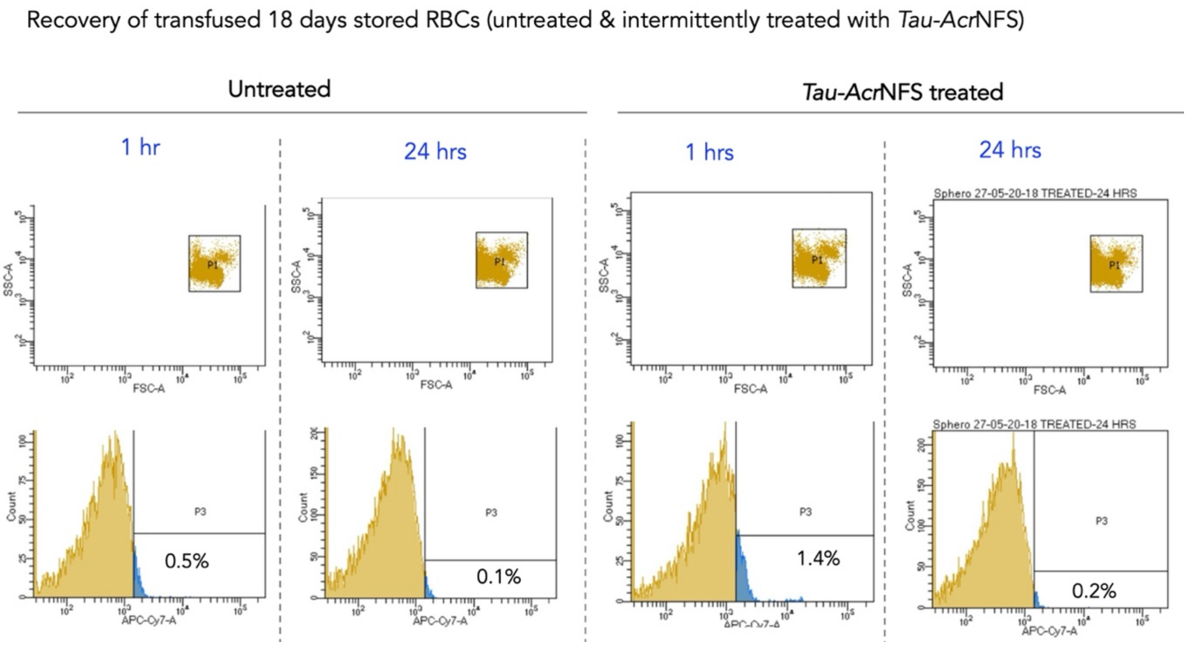

Supplementary Figure 16. Flow cytometry profiles for recovery of transfused 18 days stored RBCs that were either untreated or treated intermittently with *Tau-Acr*NFS. In both groups, <25% of transfused RBCs were in circulation after 24 hours suggests that irrespective of the treatment 18 days stored RBCs are not suitable for transfusion.

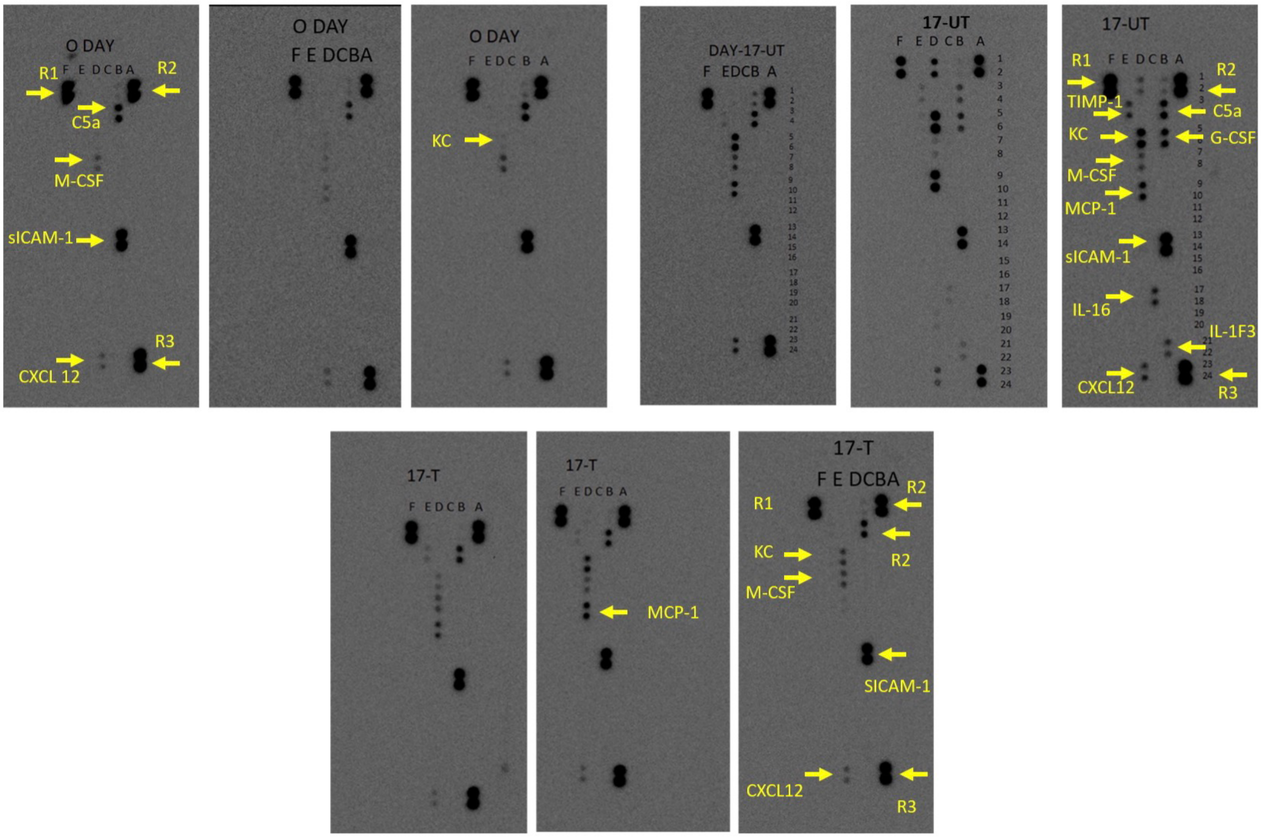

Supplementary Figure 17. Representation of cytokine array blots from all the groups (0, 14, and 17 days stored RBCs with and without the treatment. After 2 hours of transfusion, the mice were sacrificed, and the cytokines levels were measured in plasma.17D untreated (17D-UT) stored RBCs recorded elevated cytokine levels, *i.e.*, two-fold higher than the treated 17D group. The 17D UT group had elevated expression of MCP-1,CXCL12, M-CSF, KC/CXCL1 In the same group (17D untreated), increased IL-16, TIMP-1, CXCL-10, IL-1F3, G-CSF were also identified compared to untreated group.

Supplementary Table 1. The ion Q1/Q3 transitions and other parameters to quantify PUFAs.

Species	Q1 (m/z)	Q3 (m/z)	Ion Mode	DP (Volts)	EP (Volts)	CE (Volts)	CXP (Volts)
Arachidonic Acid (AA)	302.900	259.300	Negative	-209.800	-5.700	-16.200	-16.900
5-HETE	319.00	115.00	Negative	-127.00	-3.900	-18.00	-7.200
15-HETE	319.00	219.00	Negative	-158.400	-5.100	-17.200	-11.900
12-HETE	319.00	179.200	Negative	-135.500	-8.100	-19.00	-10.900
5-HETE-d_8	327.200	116.00	Negative	-127.00	-3.900	-18.00	-7.200

Supplementary Table 2. The optimized operating conditions used for ICP-MS.

Parameter	Settings
RF-Power (W)	1200
Carrier gas flow (l/min)	1.2
Plasma gas flow (l/min)	15
Auxiliary gas flow (l/min)	1.0
Spray chamber	Water cooled double pass
Spray chamber temperature (°C)	5.0
Lens voltage	4.5
Mass resolution	0.8
Integration time points/ms	3
Points per peak	3
Replicates	3
