# Supplementary material for: Intermittent scavenging of storage lesion from stored red blood cells by electrospun nanofibrous sheets enhances their quality and shelf-life
Source: Nat Commun. 2022 Dec 1;13:7394. doi: 10.1038/s41467-022-35269-3 (PMC9712616; doi:10.1038/s41467-022-35269-3)
Supplement: Supplementary file 3 — Reporting Summary [file 41467_2022_35269_MOESM3_ESM.pdf]

nature portfolio

Corresponding author(s): Praveen Kumar Vemula

Last updated by author(s): 2022/11/15

Reporting Summary

Nature Portfolio wishes to improve the reproducibility of the work that we publish. This form provides structure for consistency and transparency in reporting. For further information on Nature Portfolio policies, see our Editorial Policies and the Editorial Policy Checklist.

Statistics

For all statistical analyses, confirm that the following items are present in the figure legend, table legend, main text, or Methods section.

n/a	Confirmed	
☐	☒	The exact sample size (*n*) for each experimental group/condition, given as a discrete number and unit of measurement
☐	☒	A statement on whether measurements were taken from distinct samples or whether the same sample was measured repeatedly
☐	☒	The statistical test(s) used AND whether they are one- or two-sided<br>*Only common tests should be described solely by name; describe more complex techniques in the Methods section.*
☒	☐	A description of all covariates tested
☐	☒	A description of any assumptions or corrections, such as tests of normality and adjustment for multiple comparisons
☐	☒	A full description of the statistical parameters including central tendency (e.g. means) or other basic estimates (e.g. regression coefficient) AND variation (e.g. standard deviation) or associated estimates of uncertainty (e.g. confidence intervals)
☐	☒	For null hypothesis testing, the test statistic (e.g. *F*, *t*, *r*) with confidence intervals, effect sizes, degrees of freedom and *P* value noted<br>*Give P values as exact values whenever suitable.*
☒	☐	For Bayesian analysis, information on the choice of priors and Markov chain Monte Carlo settings
☒	☐	For hierarchical and complex designs, identification of the appropriate level for tests and full reporting of outcomes
☒	☐	Estimates of effect sizes (e.g. Cohen's *d*, Pearson's *r*), indicating how they were calculated

Our web collection on statistics for biologists contains articles on many of the points above.

Software and code

Policy information about availability of computer code

Data collection	Flow cytometry data were collected using BD LSR Fortessa cytometer (FACSDiva(TM), BD Biosciences) . Electron microscopy images were collected using FESEM (Carl Zeiss MERLIN VP compact model). LC-MS data was collected triple quadrupole mass spectrometer, QTRAP 5500. Iron concentration data collected using ICP-MS (Shimadzu-2030). Fluorescence data was collected using Fluro spectrophotometer (Horiba Fluro log QM, France). The absorbance data for Drabkin's method was collected using Varioskan LUX Multimode Microplate Reader (ThermoFisher, Massachusetts, USA)
Data analysis	Flow cytometry data were analyzed using FlowJo v10.0.8. LC-MS analysis was performed by MultiQuantTM 3.0.3 Software (SCIEX). Contact angle measurements were done using ImageJ software (V1.53)

For manuscripts utilizing custom algorithms or software that are central to the research but not yet described in published literature, software must be made available to editors and reviewers. We strongly encourage code deposition in a community repository (e.g. GitHub). See the Nature Portfolio guidelines for submitting code & software for further information.

Data

Policy information about availability of data

All manuscripts must include a data availability statement. This statement should provide the following information, where applicable:

- Accession codes, unique identifiers, or web links for publicly available datasets
- A description of any restrictions on data availability
- For clinical datasets or third party data, please ensure that the statement adheres to our policy

All data generated or analyzed during this study are included in this published article and its supplementary information files. Source data are provided with this paper with a file name Source Data.

Human research participants

Policy information about studies involving human research participants and Sex and Gender in Research.

Reporting on sex and gender	Gender was not considered in this study. Both, male and female volunteers donated the blood samples. The observed results are not gender specific.
Population characteristics	All healthy volunteers without any known infections, male and female were 25 to 35 years of age.
Recruitment	Blood donating volunteers are not involved in this study. A circular was announced for seeking healthy volunteers to donate blood. Healthy individuals who are studying at institute, and not part of this study, were volunteered to donate the blood.
Ethics oversight	Blood was collected from healthy volunteers according to the approved protocols from the Institutional Human Ethical Committee (inStem/IEC-10/003) at the Institute for Stem Cell Science and Regenerative Medicine. An informed consent has been obtained from the participants.

Note that full information on the approval of the study protocol must also be provided in the manuscript.

Field-specific reporting

Please select the one below that is the best fit for your research. If you are not sure, read the appropriate sections before making your selection.

☒ Life sciences ☐ Behavioural & social sciences ☐ Ecological, evolutionary & environmental sciences

For a reference copy of the document with all sections, see nature.com/documents/nr-reporting-summary-flat.pdf

Life sciences study design

All studies must disclose on these points even when the disclosure is negative.

Sample size	No statistical methods were used to pre-determined sample size. We performed at least three independent biological replicates. The data was within +/- 10% variation, and sufficient to obtain statistical significant data.
Data exclusions	No data were excluded from the analyses.
Replication	All experiments were repeated at least twice with the same conclusion. The exact number of experiment replications are noted in the figure legends.
Randomization	All samples were randomly assigned to groups.
Blinding	Blood donors information was blinded to the investigators. After donating the blood, volunteers are not involved in the study. Therefore, blinding to volunteers is not required. The investigators were blinded to the group allocation during the data collection.

Reporting for specific materials, systems and methods

We require information from authors about some types of materials, experimental systems and methods used in many studies. Here, indicate whether each material, system or method listed is relevant to your study. If you are not sure if a list item applies to your research, read the appropriate section before selecting a response.

Materials & experimental systems

n/a	Involved in the study
[ ]	[x] Antibodies
[x]	[ ] Eukaryotic cell lines
[x]	[ ] Palaeontology and archaeology
[ ]	[x] Animals and other organisms
[x]	[ ] Clinical data
[x]	[ ] Dual use research of concern

Methods

n/a	Involved in the study
[x]	[ ] ChIP-seq
[ ]	[x] Flow cytometry
[x]	[ ] MRI-based neuroimaging

Antibodies

Antibodies used	1. Anti-Histone-Biotin-Monoclonal antibody from mouse (cloneH11-4)<br>2. Anti-DNA-POD- Monoclonal antibody from mouse (clone MCA-33)<br>These antibodies were used for Nucleosome Assay and ELISA, Catalogue No. 11920685001 (Cell Death Detection kit, Roche, Indianapolis, IN)
Validation	The antibodies were used in 1:20 dilution as per Instruction manual.<br>Quantification of pro-inflammatory cytokine levels in serum: Catalogue No. ARY006(R&D Systems, Minnesota, USA)<br>- Detection Antibody Cocktail, Mouse Cytokine Array Panel A (serial no. 893560)<br>The detection antibody cocktail was used in the dilution of 1:100 to the prepared samples as per instruction manual.

Animals and other research organisms

Policy information about studies involving animals; ARRIVE guidelines recommended for reporting animal research, and Sex and Gender in Research

Laboratory animals	C57BL/6J mice were used which were housed in the institute animal facility. All studies were performed with both male and female mice between 8 to 12 weeks of age. Mice were housed in facility that was subjected to 12/12 hours light/dark cycle, at a temperature of 20-25 oC and humidity of 35-65%.
Wild animals	No wild animals were used in these studies.
Reporting on sex	Sex was not considered in this study. Both, male and female mice were used.
Field-collected samples	The current study did not use utilize field-collected samples.
Ethics oversight	The experimental protocols were approved by the Institutional Animal Ethics Committee (IAEC) at the Institute for Stem Cell Science and Regenerative Medicine (INS-IAE-2020/16(R1)), and were in accordance with the guidelines from the Committee for the Purpose of Control and Supervision of Experiments on Animals (CPCSEA), Govt. of India.

Note that full information on the approval of the study protocol must also be provided in the manuscript.

Flow Cytometry

Plots

Confirm that:

- [x] The axis labels state the marker and fluorochrome used (e.g. CD4-FITC).
- [x] The axis scales are clearly visible. Include numbers along axes only for bottom left plot of group (a 'group' is an analysis of identical markers).
- [x] All plots are contour plots with outliers or pseudocolor plots.
- [x] A numerical value for number of cells or percentage (with statistics) is provided.

Methodology

Sample preparation	- RBC recovery Experiment- To detect biotinylated RBCs through flow cytometry, approximately 106 -108 RBCs (100 µL of the diluted sample) were added to 0.125 µg of streptavidin– APC- efluor® 780 Conjugate (1:20 of 0.2 mg/mL; eBioscience™). Samples were incubated in the dark for 5 min, washed with 400 µL PBS, and centrifuged at 1000 g for 4 min. The RBC pellet was resuspended in 200 µL PBS. Samples were analyzed using BD LSR Fortessa cytometer (BD biosciences, USA). The red laser was used to excite the dye (λ emission = 780 nm) with a bandpass filter of 780/60.<br><br>- PS exposure Experiment-, Labelling of stored RBCs with AV was performed by adding 5 µL of AV-Alexa Fluor™ 568 conjugate (Invitrogen, USA) in 106 erythrocytes in 100 µL Annexin binding buffer. The binding buffer was prepared by adding 140mM and 2.5 mM CaCl2 in 10mM HEPES Buffer (all from Sigma-Aldrich). After incubation on ice for 15 min, cells were diluted four times with binding buffer and analyzed on an LSR Fortessa cytometer.

Instrument	BD LSR Fortessa X-20
Software	Data collected with FACS Diva v8.0.2; Data analyzed with FlowJo v10.0.8
Cell population abundance	Biotinylated cell population abundance was determined using streptativdin-APC-eflur(R) fluorescent dye, as described above. Cell numbers were calculated by multiplying events measured by the ratio of dye tagged cells over the total cells.
Gating strategy	Samples were analyzed with BD LSRFortessa™ using BD FACSDiva™ Software until 20,000 events were acquired in the P1 gate. RBCs were identified by relative size; forward and side scatter channels (FSC and SSC) were used on a logarithmic scale. The limit of positive event for the erythrocyte population with AV-Alexa Fluor™ 568 conjugate/ streptavidin– APC- efluor® 780 Conjugate were established using control (non-biotinylated RBCs) and the unstained (Biotinylated RBCs without fluorophore) (negative) sample. The P2 gate gathers all the AV-Alexa Fluor™ 568 conjugate/ streptavidin– APC- efluor® 780 Conjugate events.

☐ Tick this box to confirm that a figure exemplifying the gating strategy is provided in the Supplementary Information.
